# List of JECT reviewers 2025

**DOI:** 10.1051/ject/2025069

**Published:** 2025-12-17

The Editorial Team would like to thank this diverse and international team of peer reviewers for reviewing at least one article for JECT over the past year.

Abdelmohsen Rizq Abdelmohsen Gabr

Cory M. Alwardt, PhD, CCP

Keith Amberman, B.S., CCP

Jennifer Baeza, MS, CCP

Mostafa Bagherinasab, MS, CP

James Bennett, MSc

Salman Pervaiz Butt, DBA MBA MSc MPHYS

Andrew Chevalier, MD

Ignazio Condello, PhD

Kyle Dana, DC, MS, CP

Nathaniel Hamid Darban, PhD, CP

Edward Morse Darling, MS, CCP

Bharat Datt, MSc, CCP, CPC, FPP, FAACP, MBA

William DeBois, MBA, BS, CCP

Filip M De Somer, ECCP, PhD

Joseph J Deptula, MPS, CCP, FPP

Hongting Diao, PhD

Sylvain Diop, MD

Molly Dreher, MA, CCP

Randal Owen Dull, MD, PhD

Adam Fernandez, DHSc, CCP

David Fisher, MS, CCP

David Fitzgerald, DHA, MPH, CCP

Kenneth Fox, MD

Larry Garrison, PhD, MBA, CCP

Farhad Gorjipour, MS

Robert C. Groom, MS, CCP

Eugene A. Hessel II, MD

Porter Hunt, PhD

Richard Issitt, DClinP, FCCP, AACP

Claire B. Jara, CCP

George Justison, CCP

Koichi Kashiwa, PhD

Toshinobu Kazui, MD, PhD

Daniel Keller, MS, CCP

Christopher Komanapalli, MD

Robert Kramer, MD

Supaporn Kulthinee, PhD

Mark T. Lucas, MPS, CCP

Kara Lung, MS, CCP

Robin G. Mauger, MS, CCP Emeritus

Annette Lisa Mazzone, PhD, Diploma of Perfusion, CCP (Aust)

Kevin H. McCusker, PhD, CCP

Erick McNair, PhD, CCP, LP, FICA 

Brian Mejak, CCP

Andrew D. Meyer, MD, MS

James R. Neal, CCP FPP

Kevin Niimi, MPS, CCP

Molly Elisabeth Oldeen, MS, CCP, FPP

Vincent Olshove, BS, CCP, CCT, FPP

Jane Ottens, B.Sc.Dip. Perf., CCP (Aust)

David A. Palmer, Ed.D., CCP

Miguel Ángel Parada-Nogueiras, PhD

Thomas John Preston, MS, CCT

Luc Puis, EBCP

Thomas Rath, MPS, CCP

James Reagor, MPS, CCP

Elisa Rodrigues Fonseca, MD

Tami Rosenthal, CCP MBA

D. Bradford Sanders, MS, CCP

Keith Andrew Samolyk, BS, RRT, CCP Emeritus, PBMS

Kenneth G. Shann, CCP

David Andrew Sidebotham, MBChB, FANZCA

Justin Roy Sleasman, MS CCP

Carrie Striker, DHEd, MPS, CCP, FPP

Nathaniel Sznycer-Taub, MD

Tomohisa Takeichi, MA, CCP

Mukta Tiwari, PhD

Cody C. Trowbridge, MPS, CCP

Nousjka P.A. Vranken, MD, PhD

Tyler Wahl, CCP

Kevin Watt, MD, PhD

Patrick W. Weerwind, PhD

Julie Wegner, PhD

